# DC24: A new density coherence functional for multiconfiguration density‐coherence functional theory

**DOI:** 10.1002/jcc.27522

**Published:** 2024-11-08

**Authors:** Dayou Zhang, Yinan Shu, Donald G. Truhlar

**Affiliations:** ^1^ Department of Chemistry Chemical Theory Center, and Minnesota Supercomputing Institute, University of Minnesota Minneapolis Minnesota USA

**Keywords:** density coherence, density functional development, density matrix, multiconfiguration nonclassical‐energy functional theory, unpaired density

## Abstract

In this study, we explored several alternative functional forms to construct more accurate and more physical density coherence (DC) functionals for multiconfiguration density‐coherence functional theory. Each functional is parameterized against the same database as used in our previous work. The best DC functional, which is called DC24, has a more physical interpretation, and—as a side benefit—it also has a mean unsigned error of 1.73 kcal/mol, which is a 9% improvement as compared to the previous functional. The article also contains a new definition of the unpaired electron density, which may be useful in other contexts as well.

## Graphical abstract

Multiconfiguration density‐coherence functional theory is a low‐cost and high‐accuracy electronic structure method that recovers dynamic correlation in inherently multiconfigurational molecules and transition states by expressing the nonclassical energy as a functional of the unpaired density computed from the one‐particle reduced density matrix in the coordinate representation. Here we propose a new, more physical expression for the unpaired density and use it to optimize a density coherence functional with improved physical interpretation and upgraded accuracy.

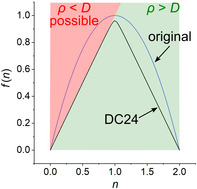

## 1 INTRODUCTION

Multiconfiguration nonclassical‐energy functional theory (MC‐NEFT)^1^, ^2^, ^3^ is a low‐cost and high‐accuracy electronic structure method to recover dynamic correlations in inherently multiconfigurational species. In MC‐NEFT, one starts with a multiconfigurational wave function, usually a multiconfiguration self‐consistent‐field (MCSCF) wave function, such as a complete active space self‐consistent field (CASSCF) wave function,^4^ that recovers the static correlation and typically a small portion of the dynamic correlation. Then, a nonclassical energy functional is used to calculate the energy from some properties of the multiconfigurational wave function. The use of a nonclassical energy functional greatly reduces the computational cost compared to traditional post‐CASSCF methods such as second‐order complete active space perturbation theory (CASPT2),^5^ multireference Møller–Plesset perturbation theory,^6^ or multireference configuration interaction^7^, ^8^ while having comparable or better accuracy in terms of bond dissociation energy,^9^, ^10^, ^11^ reaction barrier height,^12^, ^13^ excitation energies,^14^, ^15^, ^16^, ^17^, ^18^, ^19^ or other chemical properties.^20^, ^21^, ^22^, ^23^, ^24^

In recent years, we and coworkers have developed various versions of MC‐NEFT, including multiconfiguration pair‐density functional theory (MC‐PDFT),^1^, ^3^ multiconfiguration density‐coherence functional theory (MC‐DCFT),^3^, ^25^, ^26^ and multiconfiguration data‐driven functional methods (MC‐DDFMs),^2^, ^3^ including hybrid versions of MC‐PDFT^27^ and MC‐DCFT.^26^ The ultimate goal of these theories is to achieve chemical accuracy at a cost that is affordable even for complex systems.

MC‐DCFT is the most recently developed version of MC‐NEFT. It is motivated by the relationship of the off‐diagonal elements of the one‐body reduced density matrix (1‐RDM) to multiconfigurational effects in electronic wave functions,^28^ and it has an advantage of only using the 1‐RDM in the nonclassical energy functional, which in the context of MC‐DCFT is called the density coherence (DC) functional. In contrast, the two‐body reduced density matrix (2‐RDM) is needed in the nonclassical energy functional of MC‐PDFT. The reason why one uses the 1‐RDM is that it has a physical relation to the number of unpaired electrons, but we should keep in mind that the number of unpaired electrons is an interpretive quantity that has no unique definition, which will be an important consideration in the present work. The use of only the 1‐RDM not only improves the physical interpretation and reduces the computational cost, but also it simplifies the development of the theory, making it easier to extend to more complex calculations. In the present article, we present a new functional form for the DC functional and parameterize a hybrid DC functional against the same database of bond dissociation energies and reaction barrier heights as used for our previous^26^ hybrid DC functional. The new functional will be called DC24. The new functional form improves both the accuracy and the physical interpretability of the functional.

## 2 FUNCTIONAL FORMS OF DC FUNCTIONALS

The total electronic energy of hybrid MC‐DCFT (HMC‐DCFT) has the following form^25^, ^26^:

(1) $$E_{\mathrm{HMC}-\text{DCFT}}=E_{\text{class}}+XE_{\mathrm{MC},\mathrm{XC}}+\left(1-X\right)E_{\mathrm{DC},}$$

where $E_{\text{class}}$ is the classical energy from the multiconfigurational wave function, $E_{\mathrm{MC},\mathrm{XC}}$ is the MCSCF exchange–correlation energy, $E_{\mathrm{DC}}$ the nonclassical energy calculated from a DC functional, and X is a parameter. Details of $E_{\text{class}}$ and $E_{\mathrm{MC},\mathrm{XC}}$ are given in our earlier papers on MC‐DCFT^25^, ^26^; here it suffices to note that $E_{\text{class}}$ is the sum of the MCSCF kinetic energy, electron–nuclear attraction, and classical Coulomb interaction of the electronic charge distribution, and $E_{\mathrm{MC},\mathrm{XC}}$ is defined such that the sum of $E_{\text{class}}$ and $E_{\mathrm{MC},\mathrm{XC}}$ is the original CASSCF energy evaluated by the wave function variational principle. Next, we explain $E_{\mathrm{DC}}$.

In the previous versions of MC‐DCFT,^25^, ^26^ the DC functional is converted from exchange–correlation functionals used in Kohn–Sham theory. To do this, we define effective spin densities ${\tilde{\rho }}_{a}\left(r\right)$ and ${\tilde{\rho }}_{b}\left(r\right)$ using the following Equations:

(2) $${\tilde{\rho }}_{a}\left(r\right)=\frac{1}{2}\rho \left(r\right)+\frac{1}{2}D\left(r\right),$$

(3) $${\tilde{\rho }}_{b}\left(r\right)=\frac{1}{2}\rho \left(r\right)-\frac{1}{2}D\left(r\right),$$

where $\rho \left(r\right)$ and $D\left(r\right)$ are the total electron density and the unpaired electron density at a point **r** in space. We then evaluate $E_{\mathrm{DC}}$ from ${\tilde{\rho }}_{a}\left(r\right)$ and ${\tilde{\rho }}_{b}\left(r\right)$ using Kohn–Sham exchange–correlation functionals. We used the following definition for the unpaired density^29^, ^30^, ^31^, ^32^, ^33^, ^34^:

(4) $$D\left(r\right)=2\rho \left(r\right)-\int {\left[\rho \left(r|r^{\prime }\right)\right]}^{2}\;dr^{\prime }=\sum_{i}n_{i}\left(2-n_{i}\right)\chi_{i}^{2}\left(r\right),$$

where $n_{i}$ is the occupation number of natural orbital i, and $\chi_{i}\left(r\right)$ is the magnitude of natural orbital i in the MCSCF reference wave function at **r**. Note that Equation (4) may also be written as

(5) $$D\left(r\right)=\sum_{i}f\left(n_{i}\right)\chi_{i}^{2}\left(r\right),$$

(6) $$f\left(n\right)=n\left(2-n\right).$$

The function $f\left(n\right)$ can be interpreted as the effective number of unpaired electrons.^35^

Since the total density $\rho \left(r\right)$ may also be evaluated from the natural orbital and natural orbital occupation number using

(7) $$\rho \left(r\right)=\sum_{i}n_{i}\chi_{i}^{2}\left(r\right),$$

one may encounter points in space where ${\tilde{\rho }}_{b}\left(r\right)<0$ if some of the natural orbitals have occupation numbers $0<n_{i}<1$. A negative effective density is not physical, and it can produce spurious results. To tackle this issue in our earlier versions of DC functionals, we set ${\tilde{\rho }}_{b}\left(r\right)$ and $\nabla_{r}{\tilde{\rho }}_{b}\left(r\right)$ to zero for quadrature points **r** where ${\tilde{\rho }}_{b}\left(r\right)<0$:

(8) $${\tilde{\rho }}_{b}\left(r\right)=\min\left\{\frac{1}{2}\rho \left(r\right)-\frac{1}{2}D\left(r\right),0\right\},$$

this gives derivative discontinuities at points where ${\tilde{\rho }}_{b}$ passes through zero.

While the earlier versions of DC functionals work reasonably well on a wide range of systems, the derivative discontinuities are unsatisfactory. We therefore experimented with alternative ways to define the unpaired density $D\left(r\right)$ and the effective spin densities ${\tilde{\rho }}_{a}\left(r\right)$ and ${\tilde{\rho }}_{b}\left(r\right)$, and we present an improved scheme in this report.

In **new methods 1 and 2**, we use the same definition of $D\left(r\right)$ as the original method but use alternative methods to convert $\rho \left(r\right)$ and $D\left(r\right)$ into ${\tilde{\rho }}_{a}\left(r\right)$ and ${\tilde{\rho }}_{b}\left(r\right)$.

**New method 1:** Define $D\left(r\right)$ according to Equation (4) and ${\tilde{\rho }}_{b}\left(r\right)$ according to Equation (8), but redefine ${\tilde{\rho }}_{a}\left(r\right)$ according as

(9) $${\tilde{\rho }}_{a}\left(r\right)=\rho \left(r\right)-{\tilde{\rho }}_{b}\left(r\right)$$

Compared to the original method, method 1 changes the definition of ${\tilde{\rho }}_{a}\left(r\right)$ to ensure that the total density $\rho \left(r\right)$ is the sum of the effective spin densities ${\tilde{\rho }}_{a}\left(r\right)$ and ${\tilde{\rho }}_{b}\left(r\right)$.

**New method 2:** Define $D\left(r\right)$ according to Equation (4) and redefine ${\tilde{\rho }}_{b}\left(r\right)$ according to:

(10) $${\tilde{\rho }}_{b}\left(r\right)=\frac{1}{2}\rho \left(r\right)\exp\left[-\frac{D\left(r\right)}{\rho \left(r\right)}\right].$$

Then ${\tilde{\rho }}_{a}\left(r\right)$ is defined according to Equation (9) using the newly defined ${\tilde{\rho }}_{b}\left(r\right)$. Note that Equation (10) can be expanded in a series by

(11) $$\frac{1}{2}\rho \left(r\right)\exp\left[-\frac{D\left(r\right)}{\rho \left(r\right)}\right]=\frac{1}{2}\rho \left(r\right)-\frac{1}{2}D\left(r\right)+O{\left[\frac{D\left(r\right)}{\rho \left(r\right)}\right]}^{2}.$$

Equation (11) agrees with Equation (9) through the leading two terms in a Taylor series of the exponential, but it has continuous derivatives and is always non‐negative; this is the motivation for using Equation (10).

In **new methods 3 and 4**, we use the same method as the original MC‐DCFT to convert $\rho \left(r\right)$ and $D\left(r\right)$ into ${\tilde{\rho }}_{a}\left(r\right)$ and ${\tilde{\rho }}_{b}\left(r\right)$, but we use alternative definitions of $D\left(r\right)$ by taking advantage of the fact that Equation (6) for the effective number of unpaired electrons is not unique. The effective number of unpaired electrons can be defined in various ways; by changing the definition of $f\left(n\right)$, we are able to construct alternative definitions of $D\left(r\right)$.

**New method 3:** In this method, the effective number of unpaired electrons is defined by

(12) $$f\left(n\right)=\min\left(n,2-n\right),$$

and ${\tilde{\rho }}_{a}\left(r\right)$, ${\tilde{\rho }}_{b}\left(r\right)$, and $D\left(r\right)$ are defined according to Equations (2), (3), and (5) using the newly defined $f\left(n\right)$ in Equation (12). The definition of $D\left(r\right)$ according to Equations (5) and (12) is equivalent to using the unpaired electron density matrix U of Head–Gordon.^35^ Equation (12) maintains the desirable property that a natural orbital occupancy of one indicates there is exactly one unpaired electron in the orbital.

**FIGURE 1:**
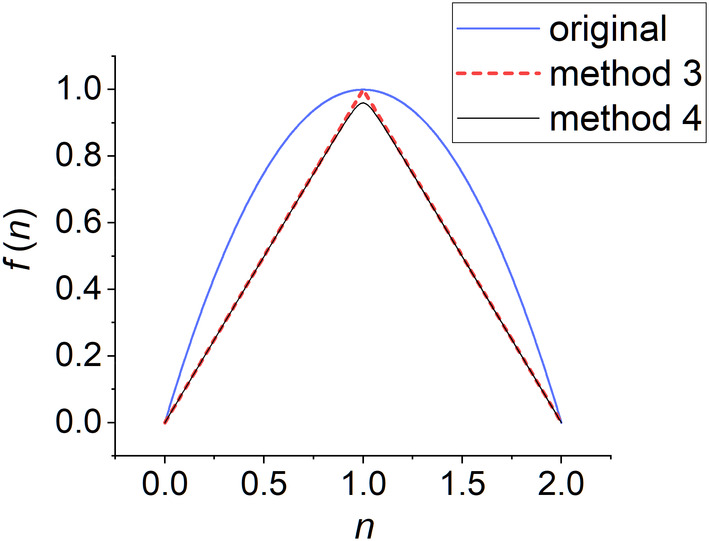
A plot of $f\left(n\right)$ of the original conversion method, method 3, and method 4 with m=0.96.

Figure 1 compares $f\left(n\right)$ of the original method and method 3. Since $f\left(n\right)\leq n$ for $f\left(n\right)$ under this definition, contribution of $D\left(r\right)$ from each natural orbital will always be less or equal to that of $\rho \left(r\right)$. This makes $D\left(r\right)\leq \rho \left(r\right)$ for all points in space. However, this definition of $f\left(n\right)$ gives discontinuities at points where n=1. Generally, the potential energy surface and wave function coefficients are smooth functions (i.e. functions with continuous first derivative) of the nuclear coordinates, except at conical intersections. Because the natural orbital occupation number is a function of the nuclear coordinates, by applying the chain rule, one can show that a non‐smooth $f\left(n\right)$ can result in non‐smooth wave function coefficients, and further result in non‐smooth potential energy surface. Because non‐smooth $f\left(n\right)$ may appear in regions that are not conical intersections, method 3 may lead to unphysical non‐smooth potential energy surfaces. An example of where the f(n) of method 3 leads to a non‐smooth potential energy surface is any trajectory path along which one of the natural orbital occupation number passes through 1. Method 4 modifies the effective number of unpaired electrons such that we obtain a smooth DC functional.

**New method 4:** Here we define the effective number of unpaired electrons as

(13) $$f\left(n\right)=\frac{1}{2}\left(-\sqrt{M^{2}+4\left(n-2\right)n}+M\right).$$

Details of the construction of $f\left(n\right)$ are given in Appendix A, where we defined

(14) $$M=m+\frac{1}{m}.$$

Again, ${\tilde{\rho }}_{a}\left(r\right)$, ${\tilde{\rho }}_{b}\left(r\right)$, and $D\left(r\right)$ are defined according to Equations (2), (3), and (5) using the newly defined $f\left(n\right)$ in Equation (13).

New method 4 is an improvement to new method 3 to remove the discontinuity at n=1. Here we simply mention two key points: (i) Equation (13) is a hyperbola that satisfies $f\left(n\right)\leq \min\left(n,2-n\right)$, which also makes $D\left(r\right)\leq \rho \left(r\right)$ for all points in space. (ii) Equation (13) has one adjustable parameter, which it is convenient to take as m because this is the value of $f\left(n\right)$ at n=1; if one sets m=1, method 4 reduces to method 3.

Below we will discuss the effect of the choice of m on the performance of the DC functional. With some trial and error, we determined that m=0.96 is the most appropriate value for practical calculations in terms of accuracy and physical interpretation.

**FIGURE 2:**
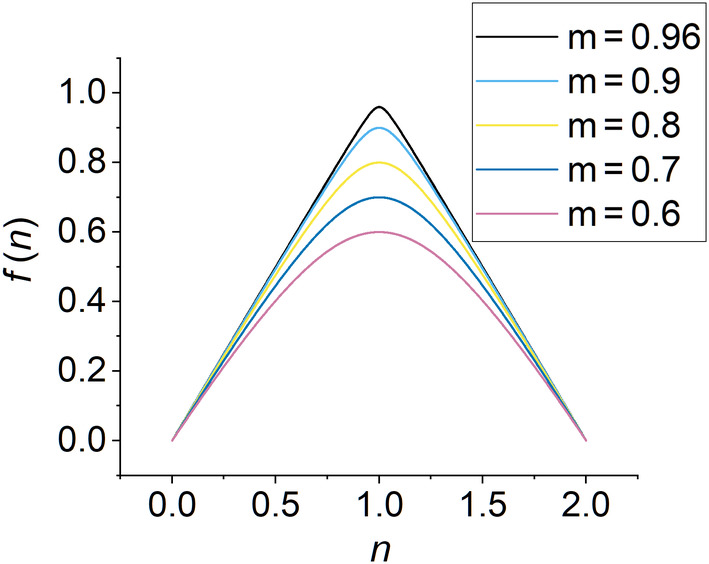
A plot of $f\left(n\right)$ of method 4 with various m values.

Figure 1 compares $f\left(n\right)$ of new method 4 with m=0.96 to $f\left(n\right)$ of new method 3, and Figure 2 compares $f\left(n\right)$ of new method 4 with various m values. As m increases, the shape of $f\left(n\right)$ becomes closer to that of method 3. When m=0.96, the shape is almost identical to that of method 3. As m continue to approach 1, a perhaps‐unwelcome consequence of this improvement in shape starts to occur; the gradient of $f\left(n\right)$ changes more rapidly at n=1. In the limit of m=1, $f\left(n\right)$ of method 4 would become identical to that of method 3, with a discontinuous derivative at n=1. We wish to avoid discontinuous or very large derivatives because they could cause numerical problems.

It is also interesting to consider the small‐*n* behavior of Equation (13). One can easily show that

(15) $$f\left(n\right)\overset{n\to 0}{\sim }\frac{2n}{M}$$

We set *m* = 0.96, which yields *M* ≈ 2.00167. Therefore

(16) $$f\left(n\right)\overset{n\to 0}{\sim }0.99917n,$$

which is quite close to the ideal behavior of 1.0*n* and which explains the initial slope seen in Figure 2.

## 3 COMPUTATIONAL DETAILS

To test the performance of DC functionals constructed using the four methods above, we parametrized hybrid DC functionals based on the HCTH^36^ functional. We used the same procedure and the same multireference database as used to parameterize the rcHCTHh functional in our previous work.^26^ The multireference database consists of 59 bond energies and 60 reaction barrier heights calculated from CASSCF wave functions using an automatic active space selection scheme. The ma‐TZVP basis set^37^, ^38^ is used for all calculations. The CASSCF calculations were performed in *Molpro* ^39^, ^40^, ^41^ or *OpenMolcas*.^42^ A Python module based on *PySCF* ^43^ and *Libxc* ^44^ was used to perform MC‐DCFT calculations. All MC‐DCFT calculations are performed with a grid with 99 radial shells and 590 angular points per shell. The parameters in the DC functionals are optimized using a Python program based on *PyTorch*,^45^ *Numpy*,^46^ and *Pandas*.^47^ The accuracy of each functional is characterized by the mean unsigned error (MUE) of the predicted bond energies and reaction barrier heights. The MUE is calculated using all 119 data points, as in our previous work.^26^

Note that we used a slightly different grid as compared to our previous work. We therefore reparametrize the rcHCTHh functional using the new grid and label it as “original.”

## 4 RESULTS AND DISCUSSIONS

The parameters of the DC functional optimized with each of the four methods and the original method are given in Table 1; the entries for method 4 are shown for *m* = 0.96 and are labeled DC24, which will be justified below. The parameters for methods other than method 4 with *m* = 0.96 are shown (at the request of a reviewer) only to show the effect of modifying the definition of *D*(**r**) on the functional parameters. We do not recommend using these parameters for applications.

**TABLE 1:** Optimized parameters of selected density coherence functionals^*a*^.

Parameter	Original	New method 1	New method 2	New method 3	DC24^b^
*C*_X*σ*,0_	8.389816E‐1	8.312883E‐1	8.884915E‐1	8.196504E‐01	8.198942E‐01
*C*_X*σ*,1_	2.759639E+0	3.176913E+0	8.111523E‐1	3.279298E+00	4.106753E+00
_X*σ*,2_	−3.648764E+1	−3.982962E+1	−1.643938E+1	−3.313177E+01	−3.716774E+01
*C*_X*σ*,3_	1.201619E+2	1.291940E+2	7.245867E+1	1.008279E+02	1.100812E+02
*C*_X*σ*,4_	−1.109976E+2	−1.189056E+2	−7.352780E+1	−8.937243E+01	−9.600026E+01
*C*_C*σσ*,0_	1.564989E+1	1.624336E+1	2.370240E+1	1.363533E+01	1.352989E+01
*C*_C*σσ*,1_	−8.579724E+1	−9.223142E+1	−1.553990E+2	−7.024657E+01	−6.881959E+01
*C*_C*σσ*,2_	3.016037E+2	3.281330E+2	5.176448E+2	2.510407E+02	2.371350E+02
*C*_C*σσ*,3_	−4.436962E+2	−4.854771E+2	−6.979786E+2	−3.692814E+02	−3.433615E+02
*C*_C*σσ*,4_	2.280074E+2	2.497807E+2	3.327389E+2	1.874674E+02	1.720927E+02
*C*_C*αβ*,0_	1.306978E+0	1.316620E+0	−7.331295E−2	1.389379E+00	1.134169E+00
*C*_C*αβ*,1_	1.683738E+1	1.713436E+1	−2.627903E+0	8.884804E+00	1.148509E+01
*C*_C*αβ*,2_	−6.727569E+1	−6.921491E+1	−9.298603E+0	−1.466935E+01	−2.210990E+01
*C*_C*αβ*,3_	6.584962E−1	−1.365050E+0	−1.190761E+2	−9.389280E+01	−1.006682E+02
*C*_C*αβ*,4_	8.021591E+1	8.666794E+1	1.452904E+2	1.291839E+02	1.477906E+02
*X*	4.631819E‐1	4.599322E‐1	5.145730E‐1	4.499314E‐01	4.525671E‐01

^a^ The parameter *X* is the scaling factor of the CASSCF exchange–correlation energy (see Equation 1). All other parameter symbols are the same as in the HCTH paper (ref 48).

^b^ New method 4 with *m* = 0.96.

The accuracy of the four new methods is compared to the original method in Table 2. The signed errors for the individual data points can be found in Table S1 in the Supporting Information. As shown in Table 2, all four methods have consistently slightly better accuracy for barrier height than bond energies. In the discussion below, we will focus on the overall MUE.

**TABLE 2:** Mean unsigned error (MUE) in kcal/mol of four new methods to construct DC functionals as compared to the original DC functional.

Method	Overall MUE	Barrier height MUE	Bond energy MUE
Original	1.91	1.79	2.03
Method 1	1.89	1.79	1.99
Method 2	2.19	1.89	2.50
Method 3	1.71	1.57	1.85
Method 4 with *m* = 0.96	1.73	1.57	1.88

Abbreviation: DC, density coherence.

In all four methods, we ensure that the total density $\rho \left(r\right)$ is the sum of the effective spin densities ${\tilde{\rho }}_{a}\left(r\right)$ and ${\tilde{\rho }}_{b}\left(r\right)$. This is the only improvement in method 1, which gives a slight improvement in the overall MUE. As a workaround to overcome the negative ${\tilde{\rho }}_{b}\left(r\right)$, method 2 does not improve the accuracy of the DC functional. In methods 3 and 4, we not only conserve the total density $\rho \left(r\right)$, but we also have a more physically well‐defined ${\tilde{\rho }}_{a}\left(r\right)$ and ${\tilde{\rho }}_{b}\left(r\right)$. All four methods have very similar errors as compared to the original method, with only minor differences, but as a side benefit of giving the effective spin densities ${\tilde{\rho }}_{a}\left(r\right)$ and ${\tilde{\rho }}_{b}\left(r\right)$ a clearer physical meaning, new methods 1, 3, and 4 have a slight improvement on the overall MUE, especially for methods 3 and 4, where a 9% decrease in overall MUE is observed.

**FIGURE 3:**
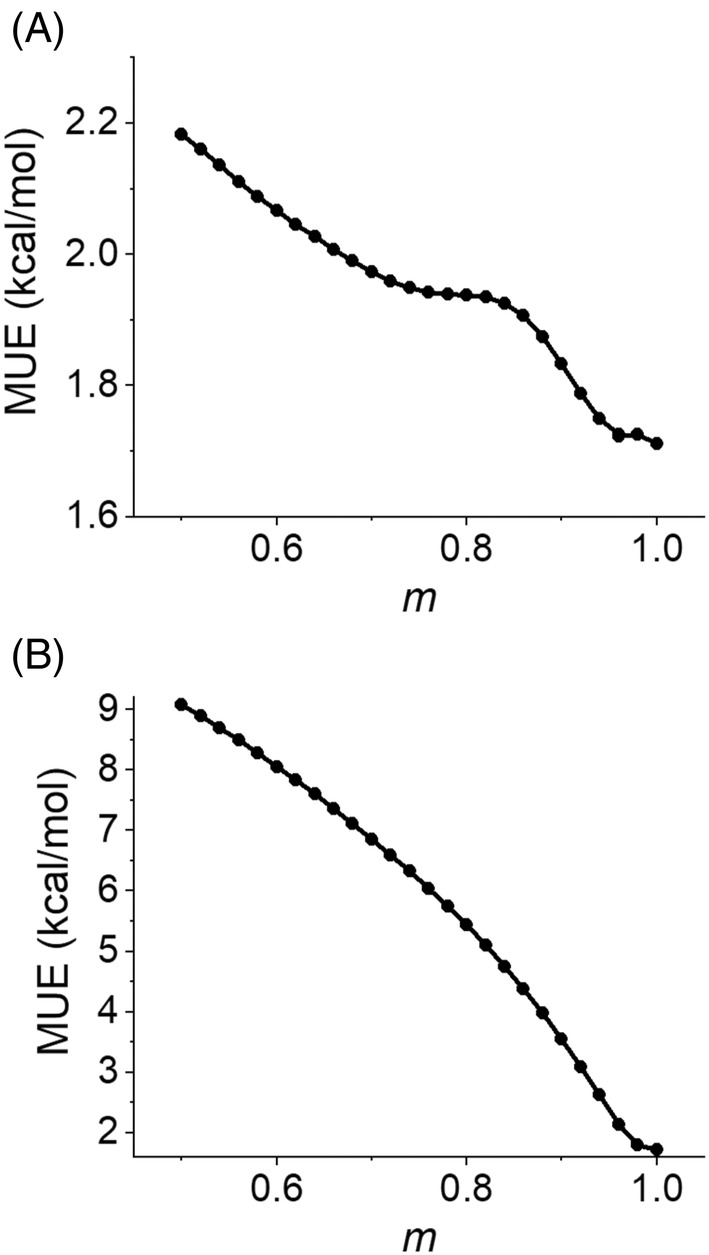
A plot of overall MUE of method 4 with various m values. The m values are sampled between 0.5 and 1.0 at 0.02 intervals. (A) Parameters are optimized for each m value. (B) Each functional uses parameters from method 3. MUE, mean unsigned error.

Figure 3A compares the overall MUE of method 4 with various m values. The functional parameters are optimized for each m value. As an overall trend, MUE decreases as m increases, with m=1 (i.e., method 3) having the lowest MUE. For practical purposes, it is desirable to have an m value that is low in MUE while having a numerically stable gradient. By analyzing Figures 2 and 3A, we empirically selected m=0.96 as the best‐compromise m value. Figure 3A shows that the MUE does not improve very much in going from *m =* 0.96 to *m* = 1, while Figure 2 shows $f\left(n\right)$ is reasonably smooth at n=1 when *m* = 0.96. We therefore chose new method 4 with *m* = 0.96 as our new functional, and we name it DC24.

For comparison purposes, at the request of a reviewer, Figure 3B shows the overall MUE for variants of method 4 in which *m* is varied, but the parameters are fixed at their values for method 3. The curve in panel (B) is smoother than that in panel (A), but the MUE is much higher at smaller m values due to the functional parameters not being consistently optimized.

## 5 CONCLUDING REMARKS

In this study, we evaluated four new functional forms to construct DC functionals. As a result of this evaluation, we presented a new functional called DC24 that supersedes our two previous DC functionals. As compared to our previous functionals, DC24 has improved accuracy and improved physical interpretation. It has a MUE of 1.73 kcal/mol over the database used for parameterization, which is a 9% improvement compared to the earlier version of the DC functional. It also has a much clearer physical meaning than the earlier version and has the advantage of having continuous functional derivative.

The development of the new functional also leads to a new expression for the unpaired electron density. Unpaired electron density is an interpretative tool of quantum chemistry, and the literature already contains multiple definitions with various pros and cons.^29^, ^30^, ^31^, ^35^, ^49^, ^50^, ^51^ The new functional form presented here, which is the combination of Equations (5) and (13), has the feature that the density at a point in space is always less than or equal to the density at that point, which is a clearly desirable constraint. This definition of the unpaired electron density may be useful in other contexts as well as for the purpose for which it is used here.

We recommend using DC24 for future DC functional applications, and it can also serve as a starting point for the development of even better DC functionals, for example by adding other ingredients like kinetic energy density, which has been very successful^52^, ^53^ in Kohn–Sham theory. While the current work provides a proof of concept of a new functional form for DC functionals, the training set is not diverse enough for broadly accurate parameterization, and more work needs to be done to develop accurate DC functionals for general applications, including designing more physical and flexible functional forms and performing functional training over a more diverse database.

## SOFTWARE AVAILABILITY STATEMENT

The development branch of *PySCF* to perform calculations with DC24 is available at https://github.com/Dayou-Zhang/pyscf-forge branch mc‐dcft. A sample input file is available in the Supporting Information.

## Supporting information


**Data S1:** Supporting Information.

## Data Availability

The data that support the findings of this study are available in the Supporting Information.

## APPENDIX A CONSTRUCTION OF *f*(*n*) IN METHOD 4

### A.1

We constructed $f\left(n\right)$ as a hyperbola that satisfies the following properties:

1. It has a similar shape as $g\left(n\right)=\min\left(n,2-n\right)$ for 0≤n≤2.
2. The asymptotic slopes are ±1.
3. It is symmetric about n=1.
4. $f\left(0\right)=f\left(2\right)=0$.
5. $f\left(1\right)=m$, where 0≤m≤1

Hyperbolas satisfying conditions (1)–(3) have the following general form:

(A1)
$$\frac{{\left(y-b\right)}^{2}}{a^{2}}-\frac{{\left(x-1\right)}^{2}}{a^{2}}=1,y\leq b,$$

where a and b are parameters to be solved. Transforming Equation (A1), we have

(A2)
$$y=f\left(x\right)=b-\sqrt{a^{2}+x^{2}-2x+1.}$$

Using conditions (4) and (5), we can solve for $a^{2}$ and b:

(A3)
$$a^{2}=\frac{{\left(m^{2}-1\right)}^{2}}{4m^{2}},$$

(A4)
$$b=\frac{1+m^{2}}{2m}.$$

This gives

(A5)
$$f\left(x\right)=\frac{1}{2}\left(-\sqrt{m^{2}+\frac{1}{m^{2}}+4\left(x-2\right)x+2}+m+\frac{1}{m}\right).$$

We can simplify this by defining

(A6)
$$M=m+\frac{1}{m}.$$

Then

(A7)
$$f\left(x\right)=\frac{1}{2}\left(-\sqrt{M^{2}+4\left(x-2\right)x}+M\right),$$

which is equivalent to Equation (13).

If we set m=1, $f\left(x\right)=1-\left|x-1\right|$, which is equivalent to $g\left(n\right)=\min\left(n,2-n\right)$. This shows that method 3 is a special case of method 4.

We can also prove that $f\left(x\right)\leq x$ for all 0≤x≤2 and 0<m≤1. The first derivative of $f\left(x\right)$ at x=0 is always less than or equal to 1, with equality if and only if m=1, as shown by the inequality of arithmetic and geometric means.

(A8)
$$f^{\prime }\left(0\right)=\frac{2}{\sqrt{m^{2}+\frac{1}{m^{2}}+2}}\leq \frac{2}{\sqrt{2\sqrt{\frac{1}{m^{2}}\cdot m^{2}}+2}}=1.$$

We can show that the second derivative of $f\left(x\right)$ is always non‐positive for all 0≤x≤2 and 0<m≤1.

(A9)
$$f^{\prime \prime }\left(x\right)=-\frac{2{\left(m^{2}-1\right)}^{2}}{\left[m^{4}+m^{2}\left(4x^{2}-8x+2\right)+1\right]\sqrt{m^{2}+\frac{1}{m^{2}}+4\left(x-2\right)x+2}}.$$

The numerator and the square root in the denominator are always non‐negative. We shall next consider the sign of the first term in the denominator. By substituting $2t=4x^{2}-8x+2$, this term can be transformed into

(A10)
$$m^{4}+m^{2}\left(4x^{2}-8x+2\right)+1=m^{4}+2{\mathrm{tm}}^{2}+1={\left(m^{2}+t\right)}^{2}+1-t^{2.}$$

Since 0≤x≤2, −1≤t≤1, and $1-t^{2}\geq 0$, which means this term is non‐negative.

Now that we have proved $f^{\prime }\left(0\right)\leq 1$ and $f^{\prime \prime }\left(x\right)\leq 0$ for all 0≤x≤2 and 0<m≤1, we have $f^{\prime }\left(x\right)\leq 1$. Since $f\left(0\right)=0$, we have $f\left(x\right)\leq x$.

By showing $f\left(x\right)\leq x$, we can further conclude that $D\left(r\right)$ defined using the new $f\left(n\right)$ satisfies $D\left(r\right)\leq \rho \left(r\right)$ for all points in space.
