# Supplementary material for: DC24: A new density coherence functional for multiconfiguration density‐coherence functional theory
Source: J Comput Chem. 2024 Nov 8;46(1):e27522. doi: 10.1002/jcc.27522 (PMC11683761; doi:10.1002/jcc.27522)
Supplement: Supplementary file 1 — Data S1: Supporting Information. [file JCC-46-0-s001.pdf]

SUPPORTING INFORMATION

OCTOBER 3, 2024

DC24: A New Density Coherence Functional for Multiconfiguration Density-Coherence Functional Theory

Dayou Zhang,† Yinan Shu,† and Donald G. Truhlar*†

†*Department of Chemistry, Chemical Theory Center, and Minnesota Supercomputing Institute, University of Minnesota, Minneapolis, Minnesota 55455-0431, United States*

*truhlar@umn.edu

TABLE OF CONTENTS

Table S1	Signed errors of selected density coherence functionals	S-2
Table S2	Effective number of unpaired electrons (ENUE) of the CASSCF wave functions	S-6
Sample input file		S-10
References		S-11

Table S1. Signed errors of selected density coherence functionals (kcal/mol)

Database[a]	Description[b]	Original	New method 1	New method 2	New method 3	DC24: New method 4 (m = 0.96)
DS1 CPO	CrH	1.61	1.14	7.49	-0.85	0.26
	MnH	2.71	2.66	3.52	3.10	2.31
	FeH	-1.24	-1.26	-1.11	-1.77	-0.52
DS1 HTBH38	$H + HCl \rightarrow H_2 + Cl$	-1.34	-1.35	-1.88	-1.86	-2.00
	V_r $H + HCl \rightarrow H_2 + Cl$	-1.97	-2.06	-1.24	-2.43	-2.62
	V_r $OH + H_2 \rightarrow H_2O + H$	-0.39	-0.33	0.11	-0.27	-0.39
	V_r $CH_3 + H_2 \rightarrow CH_4 + H$	-1.33	-1.36	-0.44	-1.34	-1.32
	$OH + CH_4 \rightarrow H_2O + CH_3$	3.45	3.34	5.75	3.45	3.42
	V_r $OH + CH_4 \rightarrow H_2O + CH_3$	0.58	0.67	0.90	0.77	0.66
	$H + H_2 \rightarrow H_2 + H$	0.38	0.44	-1.37	-0.40	-0.57
	$OH + NH_3 \rightarrow H_2O + NH_2$	2.64	2.62	2.80	2.77	2.63
	V_r $OH + NH_3 \rightarrow H_2O + NH_2$	0.71	0.79	0.71	0.58	0.29
	V_r $OH + C_2H_6 \rightarrow H_2O + C_2H_5$	0.29	0.32	0.14	0.74	0.59
	V_r $F + H_2 \rightarrow HF + H$	-2.68	-2.68	-3.37	-2.51	-2.18
	$H + PH_3 \rightarrow H_2 + PH_2$	0.44	0.46	-2.56	0.37	0.22
	V_r $H + PH_3 \rightarrow H_2 + PH_2$	-0.82	-0.84	-2.98	-1.41	-1.71
	$H + HO \rightarrow H_2 + O$	-0.09	0.02	0.42	0.02	0.26
	$H + H_2S \rightarrow H_2 + HS$	2.41	2.42	0.64	1.85	1.90
	V_r $H + H_2S \rightarrow H_2 + HS$	1.41	1.34	0.38	0.73	0.61
	$O + HCl \rightarrow OH + Cl$	7.22	7.21	8.73	7.17	7.31
	$CH_3 + NH_2 \rightarrow CH_4 + NH$	-4.16	-4.13	-4.68	-3.27	-3.16
	V_r $CH_3 + NH_2CH_4 + NH$	-2.97	-3.05	-1.82	-2.34	-2.37
	$C_2H_5 + NH_2 \rightarrow C_2H_6 + NH$	-2.37	-2.39	-2.55	-1.52	-1.30
	V_r $C_2H_5 + NH_2 \rightarrow C_2H_6 + NH$	-2.92	-2.95	-2.70	-1.70	-1.80
	$NH_2 + C_2H_6 \rightarrow NH_3 + C_2H_5$	0.95	0.89	2.21	0.95	0.97
	V_r $NH_2 + C_2H_6 \rightarrow NH_3 + C_2H_5$	0.35	0.34	-0.29	0.58	0.64
	V_r $NH_2 + CH_4 \rightarrow NH_3 + CH_3$	-0.15	-0.12	-0.75	-0.06	-0.01
DS1 MR-MGM-BE4	LiO^-	2.87	2.72	5.05	2.56	2.20
	MgS	0.50	0.81	-1.29	1.09	1.25
DS1 MR-MGN-BE17	NO	2.47	2.28	5.04	1.32	1.89
	$B_2 \rightarrow 2B$	-2.98	-3.16	-3.05	-2.12	-1.85
DS1 MR-TM-BE12	VO	-1.57	-1.77	-1.67	-1.50	-1.59
DS1 NHTBH38	$H + N_2O \rightarrow OH + N_2$	3.95	3.76	3.11	3.39	3.61
	$H + FH \rightarrow HF + H$	-1.21	-1.30	0.23	-1.75	-1.68
	$H + ClH \rightarrow HCl + H$	3.70	3.68	2.51	2.24	2.43
DS1 SR-MGM-BE8	NaO	-0.91	-0.89	-0.92	-0.25	-0.62
DS1 SR-MGN-BE107	C_2H_6	0.52	0.44	0.99	0.90	0.68
	C_2H_6O	-0.48	-0.42	-0.36	-0.25	-0.44

Database[a]	Description[b]	Original	New method 1	New method 2	New method 3	DC24: New method 4 (m = 0.96)
	Et-H	-0.04	-0.04	0.37	0.00	-0.01
	Et-CH_3	-0.04	-0.05	0.45	-0.01	-0.33
	Et-OCH_3	3.56	3.62	4.45	3.23	3.20
	Et-OH	-1.61	-1.57	-1.43	-1.51	-1.65
	CH($^2\Pi$)	1.07	1.14	-1.22	0.30	0.16
	NH	0.57	0.66	-0.85	0.18	0.25
	OH	-2.78	-2.63	-2.68	-2.58	-2.43
	HCl	-0.07	0.04	-2.17	0.04	-0.24
	Si_2 (triplet)	3.22	3.07	1.48	1.10	1.68
	P_2	-1.90	-2.03	-2.37	-2.80	-3.25
	S2	2.10	2.00	3.78	2.64	3.00
	SC	3.92	3.90	2.12	3.68	3.38
	H_2	-1.18	-1.21	-0.96	-1.20	-1.42
	SH	1.38	1.50	0.66	1.60	1.35
DS1 SR-TM-BE15	Cu_2	-1.76	-1.93	-1.88	-1.35	-1.76
	$CrCH_3^+$	-1.26	-0.97	-1.09	-0.80	-1.74
DS2 CPO	MnH	2.73	2.62	4.93	2.79	2.26
	FeH	-0.87	-0.85	0.22	-2.12	-1.12
DS2 HTBH38	H + HCl→ H_2 + Cl	-1.23	-1.21	-2.22	-1.63	-1.80
	V_r H + HCl→ H_2 + Cl	-1.06	-1.17	-0.19	-1.74	-1.90
	V_r OH + H_2→ H_2O + H	-0.39	-0.33	0.11	-0.27	-0.39
	V_r CH_3 + H_2→ CH_4 + H	-1.33	-1.36	-0.44	-1.34	-1.32
	OH + CH_4→ H_2O + CH_3	3.10	3.06	4.33	3.49	3.34
	V_r OH + CH_4→ H_2O + CH_3	0.41	0.55	-0.20	0.75	0.52
	H + H_2→ H_2 + H	0.38	0.44	-1.37	-0.40	-0.57
	OH + NH_3→ H_2O + NH_2	2.83	2.76	3.69	2.65	2.61
	V_r OH + NH_3→ H_2O + NH_2	0.76	0.85	0.79	0.69	0.39
	OH + C_2H_6→ H_2O + C_2H_5	0.50	0.46	1.30	1.15	1.02
	V_r OH + C_2H_6→ H_2O + C_2H_5	-2.53	-2.47	-2.84	-2.17	-2.31
	F + H_2→ HF + H	-0.17	-0.44	1.30	0.15	0.53
	V_r F + H_2→ HF + H	-2.68	-2.68	-3.37	-2.51	-2.18
	V_r O + CH_4→ OH + CH_3	-3.95	-3.85	-3.37	-3.31	-3.13
	H + PH_3→ H_2 + PH_2	1.90	1.87	-0.55	1.69	1.71
	V_r H + PH_3→ H_2 + PH_2	0.76	0.72	-0.98	0.12	-0.03
	H + HO→ H_2 + O	-0.58	-0.45	-0.41	-0.22	0.01
	V_r H + HO→ H_2 + O	2.08	1.99	2.94	1.62	1.49
	H + H_2S→ H_2 + HS	2.41	2.42	0.64	1.85	1.90
	V_r H + H_2S→ H_2 + HS	1.49	1.42	0.42	0.82	0.72
	V_r O + HCl→ OH + Cl	-0.49	-0.45	1.39	-0.26	0.12
	CH_3 + NH_2→ CH_4 + NH	-2.52	-2.49	-2.57	-1.80	-1.63

Database[a]	Description[b]	Original	New method 1	New method 2	New method 3	DC24: New method 4 (m = 0.96)
	V_r $CH_3 + NH_2 \rightarrow CH_4 + NH$	-2.97	-3.05	-1.82	-2.34	-2.37
	$C_2H_5 + NH_2 \rightarrow C_2H_6 + NH$	-2.37	-2.39	-2.55	-1.52	-1.30
	V_r $C_2H_5 + NH_2 \rightarrow C_2H_6 + NH$	-3.45	-3.52	-2.67	-2.24	-2.24
	$NH_2 + C_2H_6 \rightarrow NH_3 + C_2H_5$	-0.65	-0.69	0.44	-0.17	-0.13
	V_r $NH_2 + C_2H_6 \rightarrow NH_3 + C_2H_5$	-1.16	-1.15	-2.04	-0.68	-0.60
	$NH_2 + CH_4 \rightarrow NH_3 + CH_3$	0.34	0.28	2.02	0.19	0.26
	V_r $NH_2 + CH_4 \rightarrow NH_3 + CH_3$	-0.30	-0.28	-0.42	-0.26	-0.10
DS2 MR-MGM-BE4	LiO^-	-2.41	-2.54	-1.76	-2.07	-2.18
DS2 MR-MGN-BE17	SiO (singlet)	0.43	0.17	0.05	1.32	1.98
	CO	0.94	0.86	-0.37	1.78	1.68
	ClO	2.79	2.84	4.57	2.99	3.32
	$O_3 \rightarrow O_2 + O$	-5.79	-5.76	-6.37	-5.45	-5.45
	N_2	1.81	1.57	3.14	0.78	1.67
	O_2	2.40	2.24	5.94	0.95	1.03
	$B_2 \rightarrow 2B$	2.73	2.57	2.71	2.66	2.98
	$C_2 \rightarrow 2C$	-9.01	-8.62	-10.05	-9.75	-10.37
DS2 MR-TM-BE12	CuCl	-1.61	-1.84	-2.67	-1.92	-1.46
DS2 NHTBH38	$H + FH \rightarrow HF + H$	-1.21	-1.30	0.23	-1.75	-1.68
	$H + ClH \rightarrow HCl + H$	3.70	3.68	2.51	2.24	2.43
	V_r $H + FCH_3 \rightarrow HF + CH_3$	-2.19	-2.21	-2.78	-1.85	-2.11
	$H + F_2 \rightarrow HF + F$	4.67	4.39	5.02	3.98	4.97
DS2 SR-MGM-BE8	NaO	3.47	3.50	4.04	3.80	3.44
	ZnCl	-3.39	-3.52	-3.93	-3.58	-2.79
DS2 SR-MGN-BE107	C_2H_6	0.52	0.43	0.99	0.90	0.68
	C_2H_6O	5.57	5.57	6.14	5.46	5.44
	Et-H	-0.15	-0.09	-0.54	-0.10	-0.23
	Et-CH_3	-0.05	-0.05	0.45	-0.01	-0.33
	Et-OCH_3	-1.47	-1.35	-1.58	-1.39	-1.63
	Et-OH	0.42	0.51	-0.02	0.41	0.19
	$CH(^2\Pi)$	1.08	1.15	-1.18	0.28	0.13
	NH	0.18	0.26	-0.74	-0.32	-0.19
	OH	-0.30	-0.16	0.09	-0.15	0.04
	HCl	0.09	0.14	-1.09	0.17	-0.07
	Si_2 (triplet)	-5.66	-4.84	-4.51	0.39	0.33
	P_2	-1.90	-2.03	-2.37	-2.80	-3.25
	S_2	2.06	1.97	3.73	2.59	2.95
	Cl_2	-3.37	-3.21	-4.24	-1.91	-2.09
	SC	3.58	3.57	1.87	3.29	2.99
	H_2	-1.18	-1.21	-0.96	-1.20	-1.42

Database[a]	Description[b]	Original	New method 1	New method 2	New method 3	DC24: New method 4 (m = 0.96)
	SH	3.48	3.55	3.20	3.43	3.30
DS2 SR-TM-BE15	FeCl	-3.76	-3.68	-4.47	-3.82	-4.38

[a]DS1 denotes Data Set 1, for which CASSCF reference wave functions were generated by *Molpro*; DS2 denotes Data Set 2, for which CASSCF reference wave functions were generated by *OpenMolcas*. The database labeled CPO is from the corelated-participating-orbital paper (ref. 1). The other databases are from Minnesota Database 2019.[2]

[b]For reactions preceded by V_r, the energies and errors are for the reverse barrier height.

Table S2. Effective number of unpaired electrons (ENUE) of the CASSCF wave functions[a]

Database	Description	ENUE at combined structure[b]	ENUE at dissociated structure[c]
DS1 CPO	CrH	5.24	7.00
	MnH	6.02	6.29
	FeH	4.79	5.34
DS1 HTBH38	H + HCl → H_2 + Cl	1.01	1.00
	V_r H + HCl → H_2 + Cl	1.01	1.00
	V_r OH + H_2 → H_2O + H	1.00	1.00
	V_r CH_3 + H_2 → CH_4 + H	1.00	1.00
	OH + CH_4 → H_2O + CH_3	1.09	1.09
	V_r OH + CH_4 → H_2O + CH_3	1.09	1.08
	H + H_2 → H_2 + H	1.00	1.00
	OH + NH_3 → H_2O + NH_2	1.13	1.05
	V_r OH + NH_3 → H_2O + NH_2	1.13	1.03
	V_r OH + C_2H_6 → H_2O + C_2H_5	1.09	1.07
	V_r F + H_2 → HF + H	1.00	1.00
	H + PH_3 → H_2 + PH_2	1.11	1.00
	V_r H + PH_3 → H_2 + PH_2	1.11	1.08
	H + HO → H_2 + O	2.00	2.00
	H + H_2S → H_2 + HS	1.08	1.00
	V_r H + H_2S → H_2 + HS	1.08	1.05
	O + HCl → OH + Cl	2.07	2.04
	CH_3 + NH_2 → CH_4 + NH	2.24	2.16
	V_r CH_3 + NH_2CH_4 + NH	2.24	2.18
	C_2H_5 + NH_2 → C_2H_6 + NH	2.24	2.18
	V_r C_2H_5 + NH_2 → C_2H_6 + NH	2.24	2.09
	NH_2 + C_2H_6 → NH_3 + C_2H_5	1.08	1.03
	V_r NH_2 + C_2H_6 → NH_3 + C_2H_5	1.08	1.05
	V_r NH_2 + CH_4 → NH_3 + CH_3	1.08	1.05
DS1 MR-MGM-BE4	LiO^-	2.17	2.09
	MgS	0.60	2.46
DS1 MR-MGN-BE17	NO	1.47	5.05
	B_2 → 2B	3.31	2.42
DS1 MR-TM-BE12	VO	3.75	7.04
DS1 NHTBH38	H + N_2O → OH + N_2	1.01	1.00
	H + FH → HF + H	1.00	1.00
	H + ClH → HCl + H	1.00	1.00
DS1 SR-MGM-BE8	NaO	1.07	3.00
DS1 SR-MGN-BE107	C_2H_6	0.22	2.15
	C_2H_6O	0.23	2.15
	Et-H	0.15	2.08

Database	Description	ENUE at combined structure[b]	ENUE at dissociated structure[c]
	Et-CH_3	0.22	2.14
	Et-OCH_3	0.26	2.15
	Et-OH	0.24	2.09
	CH($^2\prod$)	1.35	3.18
	NH	2.16	4.02
	OH	1.10	3.00
	HCl	0.03	2.00
	Si_2 (triplet)	2.75	4.32
	P_2	0.74	6.00
	S2	2.36	4.15
	SC	0.70	4.20
	H_2	0.10	2.00
	SH	1.11	3.05
DS1 SR-TM-BE15	Cu_2	0.24	2.00
	$CrCH_3^+$	4.31	6.07
DS2 CPO	MnH	6.11	6.29
	FeH	4.85	5.44
DS2 HTBH38	H + HCl→ H_2 + Cl	1.04	1.00
	V_r H + HCl→ H_2 + Cl	1.04	1.04
	V_r OH + H_2→ H_2O + H	1.00	1.00
	V_r CH_3 + H_2→ CH_4 + H	1.00	1.00
	OH + CH_4→ H_2O + CH_3	1.09	1.01
	V_r OH + CH_4→ H_2O + CH_3	1.09	1.01
	H + H_2→ H_2 + H	1.00	1.00
	OH + NH_3→ H_2O + NH_2	1.13	1.09
	V_r OH + NH_3→ H_2O + NH_2	1.13	1.05
	OH + C_2H_6→ H_2O + C_2H_5	1.12	1.00
	V_r OH + C_2H_6→ H_2O + C_2H_5	1.12	1.07
	F + H_2→ HF + H	1.00	1.00
	V_r F + H_2→ HF + H	1.00	1.00
	V_r O + CH_4→ OH + CH_3	2.22	2.17
	H + PH_3→ H_2 + PH_2	1.10	1.00
	V_r H + PH_3→ H_2 + PH_2	1.10	1.10
	H + HO→ H_2 + O	2.04	2.01
	V_r H + HO→ H_2 + O	2.04	2.04
	H + H_2S→ H_2 + HS	1.08	1.00
	V_r H + H_2S→ H_2 + HS	1.08	1.06
	V_r O + HCl→ OH + Cl	2.20	2.14
	CH_3 + NH_2→ CH_4 + NH	2.24	2.18
	V_r CH_3 + NH_2→ CH_4 + NH	2.24	2.18
	C_2H_5 + NH_2→ C_2H_6 + NH	2.24	2.18

Database	Description	ENUE at combined structure[b]	ENUE at dissociated structure[c]
	V_r $C_2H_5 + NH_2 \rightarrow C_2H_6 + NH$	2.24	2.14
	$NH_2 + C_2H_6 \rightarrow NH_3 + C_2H_5$	1.13	1.07
	V_r $NH_2 + C_2H_6 \rightarrow NH_3 + C_2H_5$	1.13	1.07
	$NH_2 + CH_4 \rightarrow NH_3 + CH_3$	1.05	1.05
	V_r $NH_2 + CH_4 \rightarrow NH_3 + CH_3$	1.05	1.05
DS2 MR-MGM-BE4	LiO^-	2.17	2.25
DS2 MR-MGN-BE17	SiO (singlet)	0.47	4.23
	CO	0.57	4.22
	ClO	1.34	3.14
	$O_3 \rightarrow O_2 + O$	1.37	4.40
	N_2	0.65	6.05
	O_2	2.34	4.08
	$B_2 \rightarrow 2B$	3.21	2.52
	$C_2 \rightarrow 2C$	2.18	5.26
DS2 MR-TM-BE12	CuCl	0.13	2.02
DS2 NHTBH38	$H + FH \rightarrow HF + H$	1.00	1.00
	$H + ClH \rightarrow HCl + H$	1.00	1.00
	V_r $H + FCH_3 \rightarrow HF + CH_3$	1.24	1.07
	$H + F_2 \rightarrow HF + F$	1.00	1.03
DS2 SR-MGM-BE8	NaO	1.20	3.04
	ZnCl	1.05	1.23
DS2 SR-MGN-BE107	C_2H_6	0.22	2.15
	C_2H_6O	0.26	2.17
	Et-H	0.08	2.07
	Et-CH_3	0.22	2.14
	Et-OCH_3	0.22	2.15
	Et-OH	0.22	2.15
	$CH(^2\Pi)$	1.36	3.18
	NH	2.16	4.03
	OH	1.16	3.04
	HCl	0.13	2.04
	Si_2 (triplet)	2.76	5.21
	P_2	0.74	6.00
	S_2	2.36	4.15
	Cl_2	0.34	2.12
	SC	0.70	4.20
	H_2	0.10	2.00
	SH	1.18	3.05
DS2 SR-TM-BE15	FeCl	5.24	5.51

[a]The ENUE values listed in this table are defined as

$$\sum_i n_i(2 - n_i)$$

where n_i is the occupation number of natural orbital i of the CASSCF wave function.

[b]Equilibrium structures for bond energies and transition state structures for barrier heights

[c]Supermolecule of dissociated fragments for bond energies and supermolecule of reactants or products at large separation for barrier heights

Sample input file

The following *PySCF* input file performs a single-point energy calculation of H_2 with DC24. To run MC-DCFT calculations in *PySCF*, one needs to first install the latest standard version of *PySCF*, then install a development branch of *PySCF Forge* from https://github.com/Dayou-Zhang/pyscf-forge branch `mc-dcft`.

```
from pyscf import scf, gto, mcdcft

mol = gto.M(atom='H 0 0 0; H 0 0 0.74', basis='def2-tzvp',
      symmetry=False, verbose=3, unit='angstrom')
mf = scf.RHF(mol)
mf.kernel()
mc = mcdcft.CASSCF(mf, 'DC24', 2, 2, grids_level=(99, 590))
mc.chkfile = 'H2_DC24.chk'
mc.kernel()
```

References

[1] J. L. Bao, S. O. Odoh, L. Gagliardi, D. G. Truhlar, *J. Chem. Theory Comput.* **2017**, *13*, 616−626. https://doi.org/10.1021/acs.jctc.6b01102

[2] P. Verma, D. G. Truhlar, Geometries for Minnesota Database 2019. Data Repository for the University of Minnesota, **2019**. https://doi.org/10.13020/217y-8g32
